# BK polyomavirus-associated progressive multifocal leukoencephalopathy following mogamulizumab therapy for erythrodermic mycosis fungoides

**DOI:** 10.3389/fcimb.2026.1733473

**Published:** 2026-02-04

**Authors:** Michele Longo, Fabiana Napolitano, Rosy D’Agostino, Ilaria Cappuccio, Ugo de Martino, Antonio Esposito, Lorenzo Esposito, Serena Molino, Cinzia Valeria Russo, Nicola Simeone, Stefano Brusa, Rosanna Sorrentino, Luca Vallefuoco, Alessandro Severino, Fiore Manganelli, Fabrizio Pane, Giuseppe Portella

**Affiliations:** 1 Department of Translational Medicine, Federico II University of Naples, Naples, Italy; 2 Department of Neurosciences, Reproductive, and Odontostomatological Sciences, Federico II University of Naples, Naples, Italy; 3 Department of Clinical Medicine and Surgery, Federico II University of Naples, Naples, Italy

**Keywords:** BK polyoma virus, mogamulizumab, NCCR arrangements, neurotropism, PML - progressive multifocal leucoencephalopathy, VP1 analysis

## Abstract

**Introduction:**

BK polyomavirus (BKPyV) is a ubiquitous human pathogen that typically causes nephropathy and hemorrhagic cystitis in immunocompromised patients. Although BKPyV shares close genetic and structural similarity with JC polyomavirus (JCPyV), which is responsible for progressive multifocal leukoencephalopathy (PML), its neurotropic potential remains poorly characterized. Rare reports have suggested possible central nervous system (CNS) involvement under conditions of severe immune suppression. Here, we describe the first documented case of BKPyV-associated PML in a patient with erythrodermic mycosis fungoides treated with Mogamulizumab, a CCR4-targeting monoclonal antibody that profoundly alters immune surveillance.

**Results:**

We describe a patient with erythrodermic mycosis fungoides and long-standing immunological frailty, who developed neurological symptoms during Mogamulizumab therapy. Brain MRI showed multifocal white matter lesions compatible with PML. BKPyV DNA was detected in plasma, urine, and cerebrospinal fluid (CSF), while JCPyV DNA was absent. Serological testing showed high anti-BKPyV and anti-JCPyV IgG levels in plasma, indicating prior exposure to both viruses, while antibodies were undetectable in CSF, consistent with lack of intrathecal synthesis. This compartmental dissociation between plasma and CSF, together with the detection of BKPyV DNA and the absence of JCPyV DNA in CSF, supports BKPyV as the etiological neurotropic agent responsible for leukoencephalopathy. Sequencing of the VP1 and NCCR regions revealed compartment-specific nucleotide and amino acid variants, including non-conservative substitutions in the CSF isolate, suggesting intra-host viral heterogeneity. Compartment-specific sequence variability of viral protein 1 (VP1) and structural rearrangements of the non-coding control region (NCCR), particularly the loss of the Q and R block in CSF-derived isolates, underscore intra-host heterogeneity of BKV and may contribute to its adaptation and neurotropic potential.

**Conclusion:**

This is the first documented case of BKPyV-associated PML in a Mogamulizumab-treated patient. These findings highlight intra-host heterogeneity at the protein level, possibly reflecting compartment-specific viral evolution, and underscore the need for vigilant BKPyV and JCPyV monitoring during Mogamulizumab treatment.

## 1 Introduction

Polyomaviruses are circular double-stranded DNA (dsDNA) viruses. Thirteen polyomaviruses are classified as human polyomaviruses (HPyV); eleven routinely infect humans, however only six are associated with human diseases, the most relevant being BK polyomavirus (BKPyV), JC polyomavirus (JCPyV) and Merkel cell polyomavirus (MCPyV) (Cook, 2016).

BKPyV (HPyV1) is known as the etiological agent of polyomavirus nephropathy in kidney transplant patients and of hemorrhagic cystitis in hematopoietic stem cell transplant (HSCT) recipients (Aldiwani et al., 2019; Kant et al., 2022; McIlroy et al., 2019). MCPyV is responsible for a form of Merkel cell carcinoma (Feng et al., 2008).

JC virus is commonly associated with central nervous system (CNS) tropism, causing a progressive multifocal leukoencephalopathy (PML) (Haley and Atwood, 2017), a serious adverse event for some immunosuppressive therapies (Cook, 2016; Cortese et al., 2021).

Immunosuppression plays a crucial role in BKPyV and JCPyV pathology, leading to the reactivation of the infection, although with great heterogeneity ranging from asymptomatic replication to organ dysfunction such as polyomavirus nephropathy, or even a lethal outcome such as PML. Only in a minority of cases BKPyV and JCPyV reactivations lead to overt pathology (Hirsch et al., 2002).

In immunosuppressed patients, BKPyV reactivation commonly leads to nephropathy or hemorrhagic cystitis, less frequently BKPyV has been reported to cause fatal pneumonia, retinitis, native kidney nephritis (Brennan et al., 2005).

BKPyV meningoencephalitis in severely immunocompromised patients is a very rare event, and BKPyV tropism for the brain is poorly understood (Friedman and Flanders, 2006). Few cases of BKPyV encephalitis were reported in HSCT recipients (Friedman and Flanders, 2006; Yamaguchi et al., 2024).

A study detected in the cerebrospinal fluid (CSF) of children with clinically diagnosed meningitis and/or encephalitis of unknown etiology, BKPyV and MCPyV in two and four samples, respectively (Launes et al., 2024).

In a study of 2,062 CSF samples from patients with neurological disorders, BKPyV was detected in 20 patients diagnosed with PML or multiple sclerosis (Bárcena-Panero et al., 2012). CSF samples from BKPyV-positive patients were also screened for other neurotropic viruses, including JCPyV, herpes simplex virus (HSV), Epstein–Barr virus (EBV), cytomegalovirus (CMV), and human enteroviruses (EVs). Among these, one patient tested positive for JCPyV, one for HSV, one for EBV, and one for both EBV and CMV.

Mycosis fungoides is the most common form of cutaneous T-cell lymphoma and can progress to advanced stages such as the erythrodermic variant, which is often resistant to conventional therapies. In recent years, Mogamulizumab, a monoclonal antibody targeting the receptor C-C chemokine receptor 4 (CCR4) receptor, has emerged as a novel therapeutic option, although associated with a risk of immunosuppression (Whittaker et al., 2016).

Herein we present a fatal case of PML associated with BKPyV in a 66-year-old male patient diagnosed with erythrodermic mycosis fungoides receiving Mogamulizumab.

## 2 Materials and methods

### 2.1 Sample collection and storage

Urine, plasma, and cerebrospinal fluid (CSF) samples were collected, aliquoted, and stored at −80 °C until nucleic acid extraction.

### 2.2 DNA extraction and real-time PCR assays

Real-time assays for BKPyV, JCPyV, HSV, EBV, and human herpesvirus 6 (HHV-6) DNA were performed using the AltoStar PCR 1.5 (Altona Diagnostics, Hamburg, Germany) according to the manufacturer’s instructions. Nucleic acid extraction was carried out using the AltoStar AM16 automated system, and real-time PCR was performed on the Bio-Rad CFX96™ Deep Well Dx System platform.

For CMV DNA quantification, the Abbott RealTime CMV assay (Abbott Molecular, USA) was used, based on real-time quantitative PCR technology. Automated extraction was performed on the Abbott m2000sp system, and amplification was carried out on the Abbott m2000rt instrument.

### 2.3 Serological analysis

Anti-BKPyV IgG and anti-JCPyV IgG were assessed in plasma and CSF using a commercial ELISA kit (VIDIA, Czech Republic). Results were expressed as a Positivity Index (PI) according to the manufacturer’s instructions.

### 2.4 Genetic analysis of BKPyV and compartment-specific variants

Two regions of the BKPyV VP1 gene, corresponding to the first 191 amino acids of the capsid protein, and the non-coding control region (NCCR) were amplified by PCR using primer pairs previously described (12–13). PCR reactions were performed with AmpliTaq Gold DNA polymerase (Applied Biosystems) according to the manufacturer’s instructions.

PCR products were cloned into the pGEM-T Easy vector (Promega). Plasmid DNA was purified using a Qiagen miniprep kit, and inserts were Sanger sequenced in both forward and reverse directions using SP6 and T7 primers on an AB 3500 Genetic Analyzer (Applied Biosystems) with BigDye Terminator v3.1 chemistry. VP1 and NCCR sequencing was performed on urine, plasma, and CSF samples collected at the time of first virological characterization. VP1 and NCCR sequences were analyzed and compared to the BKPyV Dunlop reference strain (NC_001538.1) used for VP1 and NCCR genotyping Multiple sequence alignments were performed to detect compartment-specific variants across urine, plasma, and CSF.

Alignments were generated in R (version 4.5.1) using the msa (method = “ClustalW”) and Biostrings packages (Bioconductor), with a custom script developed to align four sequences simultaneously. The custom R script for multiple sequence alignment is available upon request.

### 2.5 Ethics statement

Ethical approval was not required for this study as it reports results obtained from routine clinical testing and diagnostic procedures performed to determine the cause of progressive multifocal leukoencephalopathy. No additional interventions were conducted beyond standard clinical care. The study was conducted in accordance with institutional and national ethical standards. Written informed consent was obtained from the patient for participation in the study. All data were fully anonymized and cannot be traced back to the individual.

## 3 Results

### 3.1 Case presentation

The patient was initially diagnosed with erythrodermic mycosis fungoides. Clinical examination and instrumental assessments did not reveal any visceral or nodal involvement. The disease was refractory to first-line therapy, which consisted of methotrexate and interferon-alpha2a.

**Figure 1:**
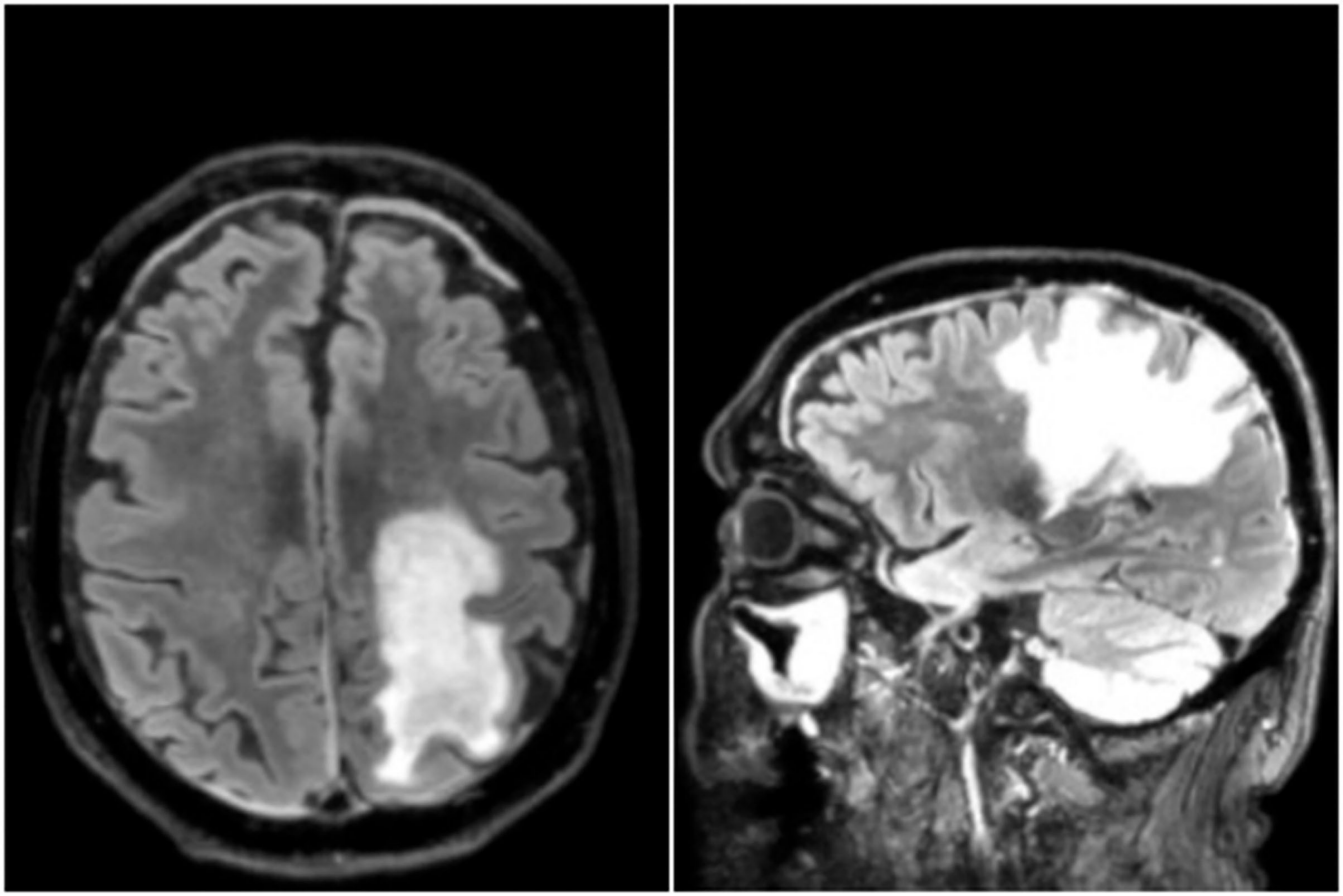
Axial (left) and sagittal (right) T2-FLAIR MRI showing a white matter hyperintensity in the left fronto-parietal region.

A significant aspect of the patient’s history was a pre-existing condition of immunological frailty, characterized by frequently recurring respiratory infections and severe bronchiectasis. Hematological and biochemical parameters at two-time point are reported in **Supplementary Table 1**.Due to the resistance to first-line treatment, the patient started Mogamulizumab therapy, with disease control for approximately one year. One year after starting Mogamulizumab treatment, the patient developed a progressive neurological impairment, characterized by cognitive and motor deficits that led to admission in the Neurology Unit. Neurological examination revealed cognitive slowing, dysarthria and right faciobrachio-crural hemiparesis and autonomous gait was impossible. The patient underwent brain magnetic resonance image (MRI) that showed multifocal white matter hyperintensities involving the left fronto-parietal region, the left internal capsule and ipsilateral cerebral peduncle as well as the paraventricular areas bilaterally and the corpus callosum (**Figure 1**). Based on such findings along with medical history, a diagnosis of PML was postulated. Accordingly, the patient underwent lumbar puncture for searching JC polyomavirus (JCPyV) in CSF, but CSF analysis resulted negative. However, since JCPyV DNA was detected in urine (3,383,920 IU/mL) and plasma (155 IU/mL), another CSF sample was analyzed and again was negative for JCPyV DNA (**Table 1**). CSF was also negative for Herpes-Simplex Virus 1 (HSV-1), Herpes- Simplex Virus 2 (HSV-2), Varicella-Zoster Virus (VZV), HHV-6, Parvovirus B19, EBV, Adenovirus, CMV and for bacteria (**Table 2**).

**Table 1:** Quantitative detection of JCPyV DNA in plasma, urine and CSF.

Pathogen/Target (DNA)	Specimen	Result (IU/mL)	Result (cps/mL)
JCPyV	Plasma	155 IU/mL	956 Cps/mL
JCPyV	Urine	3,383,920 IU/mL	20,878,786 Cps/mL
JCPyV	CSF	Not detected	Not detected

JCPyV DNA was quantified by Real-time quantitative PCR, as detailed in methods. JCPyV DNA was detected at low levels in plasma (155 IU/mL; 956.35 Cp/mL) and at high concentrations in urine (3,383,920 IU/mL; 20,878,786.4 Cp/mL), while it was undetectable in CSF. “Not detected” indicates values below the assay limit of detection. CSF: cerebrospinal fluid; IU/mL: International Units per milliliter; Cps/mL: copies per milliliter.

**Table 2:** Viral DNA testing in blood and CSF. Real-time quantitative PCR was used to quantify all targets, as detailed in Methods.

Pathogen/Target (DNA)	Specimen	Result
CMV	Whole blood	116 IU/mL
CMV	CSF	Not detected
EBV	Plasma	Not detected
EBV	CSF	Not detected
Parvovirus B19	Plasma	Not detected
Parvovirus B19	CSF	Not detected
HSV-1/2	Plasma	Not detected
HSV-1/2	CSF	Not detected
VZV	Plasma	Not detected
VZV	CSF	Not detected
HHV-6A/B	Plasma	Not detected
HHV-6A/B	CSF	Not detected

CMV DNA was detected only in whole blood (116 IU/mL) and was undetectable in CSF. EBV, parvovirus B19, HSV-1/2, VZV, and HHV-6A/B DNA were not detected in plasma or CSF. “Not detected” indicates values below the assay limit of detection. CSF: cerebrospinal fluid; CMV: cytomegalovirus; EBV: Epstein–Barr virus; HSV: herpes simplex virus; VZV: varicella-zoster virus; HHV-6: human herpesvirus 6.

CMV DNA was detected at low copy number in whole blood but not in CSF, and CSF culture was negative for bacteria (**Table 2**).

Given the strong suspicion of PML, CSF was also analyzed for BKPyV DNA, which tested positive (1,460 IU/mL). BKPyV was also detected in plasma (51,200,000 IU/mL) and urine (667 IU/mL) samples (**Table 3**). Anti- BKPyV IgG and anti-JCPyV IgG were measured in both plasma and CSF; the positivity index (PI, positive if >1.10, negative if <0.90) was positive in plasma (BKPyV: 6.57; JCPyV: 11.64) and negative in CSF (BKPyV: 0.38; JCPyV: 0.17).

**Table 3:** Detection of BKPyV DNA in plasma, urine and CSF at different time points.

Date	Pathogen/Target(DNA)	Specimen	Result (IU/mL)	Result (cps/mL)
17/12/2024	BKPyV	Urine	51,200,000 IU/mL	44,032,000 Cps/mL
	BKPyV	Plasma	667 IU/mL	573 Cps/mL
	BKPyV	CSF	1,306 IU/mL	1,123 Cps/mL
27/12/2024	BKPyV	Urine	27,560,000 IU/mL	23,701,600 Cps/mL
	BKPyV	Plasma	958 IU/mL	823 Cps/mL
	BKPyV	CSF	1,460 IU/mL	1,255 Cps/mL
07/01/2025	BKPyV	Plasma	1,774 IU/mL	1,525 Cps/mL
	BKPyV	CSF	337 IU/mL	290 Cps/mL

BKPyV DNA was assessed by Real-time quantitative PCR, as described in methods. Urine samples showed persistently high viral loads, while plasma and CSF contained substantially lower levels of BKPyV DNA at different time points. CSF: cerebrospinal fluid; IU/mL: International Units per milliliter; Cps/mL: copies per milliliter.

Accordingly, a diagnosis of BKPyV-associated PML was made and the patient started treatment with intravenous immunoglobulin (IVIg; 1 g/kg/day once, followed by 0.4 g/kg/day every 3 weeks for 3 times) and Cidofovir (1 mg/kg once weekly) along with probenecid.

Despite the treatment both cognition and motor functions rapidly declined, and the patient died because ab-ingestis pneumonia due to dysphagia after 5 months from the onset of the first symptoms.

### 3.2 Genotypic and intra-host variability of BKPyV viral protein 1 in urine, plasma, and CSF samples

To analyze the specific BKPyV sequence in the context of its unique neuronal tropism, and to determine the BKPyV subgroup, two regions of the VP1 gene were amplified and sequenced from urine, plasma, and CSF samples, as previously described (Bohl et al., 2005; Morel et al., 2017). Both assays confirmed the presence of BKPyV and allowed genotyping through comparison with reference sequences available for subtype classification.

Sequence analysis of both VP1 regions consistently identified the viral strain as BKPyV genotype II across all compartments. Although the subtype assignment was concordant, minor nucleotide differences were detected among the sequences obtained from the different samples. These findings support the existence of compartment-specific viral variants, consistent with a degree of intra-host genetic heterogeneity.

The amplified region, which encodes the first 191 amino acids of the VP1 capsid protein, was used to evaluate sequence variability by comparison with the BKPyV Dunlop reference strain (NC_001538.1) across all three compartments. Each compartment displayed a common set of nucleotide substitutions when compared to the reference sequence (**Supplementary Figure 1**). All Dunlop strain comparisons and compartment-specific variants are summarized in **Table 4**. Pairwise comparison of the three compartments revealed intra-host nucleotide heterogeneity, defined by three discriminating mutations: a G412C substitution distinguishing urine from plasma and CSF; a C186A substitution, and a G172_173insGGG insertion, which were exclusively detected in the CSF.

**Table 4:** VP1 sequence variations detected across body compartments.

Nucleotide change	Plasma	Urine	CSF	Aminoacid change	Mutation type	Compartment specificity
T135A	✓	✓	✓	–	Silent	Shared
C153T	✓	✓	✓	–	Silent	Shared
G172_173insGGG	–	–	✓	+G57 insertion	Insertion	CSF-specific
A183T	✓	✓	✓	E61D	Conservative	Shared
C186A	–	–	✓	N62K	Non-cons.	CSF-specific
T197A	✓	✓	✓	F66Y	Conservative	Shared
G212C	✓	✓	✓	S71T	Conservative	Shared
A224C	✓	✓	✓	D75A	Non-cons.	Shared
A229G	✓	✓	✓	–	Silent	Shared
G230A	✓	✓	✓	S77D	Non-cons.	Shared
G246C	✓	✓	✓	E82D	Conservative	Shared
G248A	✓	✓	✓	R83K	Conservative	Shared
C261T	–	✓	✓	–	Silent	Urine/CSF
C285A	✓	✓	✓	–	Silent	Shared
C288G	✓	✓	✓	–	Silent	Shared
T295C	✓	✓	✓	–	Silent	Shared
G327A	✓	✓	✓	–	Silent	Shared
C349A	✓	✓	✓	Q117K	Conservative	Shared
A399C	✓	✓	✓	–	Silent	Shared
G408T	✓	✓	✓	–	Silent	Shared
G412C	–	✓	–	E138Q	Conservative	Urine-specific
C415A	✓	✓	✓	H139N	Conservative	Shared
A426C	✓	✓	✓	–	Silent	Shared
A433G	✓	✓	✓	–	Silent	Shared
T435C	✓	✓	✓	I145V	Conservative	Shared
A465G	✓	✓	✓	–	Silent	Shared
A474C	✓	✓	✓	E158D	Conservative	Shared
G510A	✓	✓	✓	–	Silent	Shared
T511A	✓	✓	✓	S171T	Conservative	Shared
T522A	✓	✓	✓	–	Silent	Shared
G523C	✓	✓	✓	–	Silent	Shared
T525A	✓	✓	✓	D175Q	Non-cons.	Shared
A549T	✓	✓	✓	–	Silent	Shared
C555T	✓	✓	✓	–	Silent	Shared

Nucleotide substitutions and amino-acid changes identified in VP1 sequences obtained from plasma, urine, and cerebrospinal fluid (CSF) were compared with the BKPyV Dunlop reference strain. Check marks indicate the presence of each variant in the respective compartment. Nucleotide changes are reported at the cDNA level, with numbering based on the VP1 open reading frame (+1 corresponding to the adenine of the ATG start codon). Variants are listed according to their position in the VP1 protein sequence, with numbering relative to the first methionine of the open reading frame. Amino-acid substitutions are reported using the single-letter code. “Silent” indicates synonymous nucleotide substitutions not resulting in amino-acid changes. Conservative substitutions preserve amino-acid chemical properties, whereas Non-cons. (non-conservative) substitutions involve changes in physicochemical properties potentially affecting protein function. Insertions refer to in-frame amino-acid additions. Compartment specificity summarizes whether each variant is shared or restricted to one or more compartments.

Amino acid translation of the sequenced VP1 region revealed several substitutions (**Table 4**) compared to the BKPyV Dunlop reference (Sequences alignment shown in **Supplementary Figure 2**). Most changes were conserved across all compartments, further supporting the assignment to genotype II. However, some non-conservative substitutions showed a compartment specific distribution (**Table 4**). E138Q mutation was exclusive to the urine. Additionally, a glycine insertion at position 57 and N62K change were identified only in the CSF, mirroring the nucleotide insertion G172_173insGGG and C186A substitution. These findings highlight intra-host heterogeneity at the protein level, possibly reflecting compartment-specific viral evolution. In addition to the G57 insertion identified, non-conservative substitution N62K was detected in CSF, suggesting potential structural or functional divergence of VP1 in different anatomical sites. These findings further indicate protein-level heterogeneity across compartments, consistent with intra-host variation of VP1.

**Figure 2:**
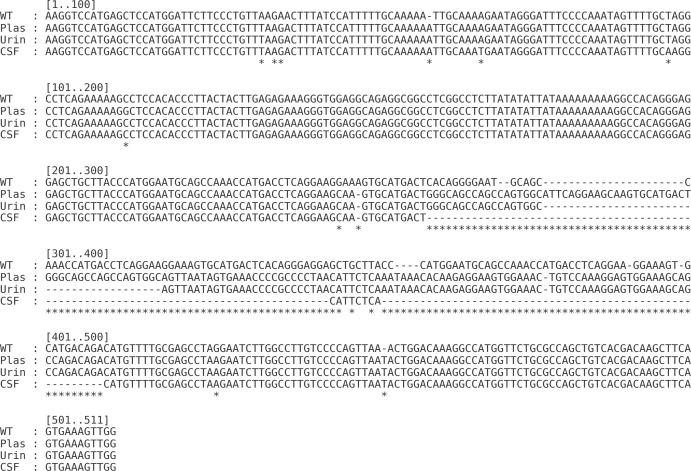
Multiple sequence alignment of BKPyV NCCR from wild-type Dunlop reference and compartment-derived isolates. Alignment of the NCCR sequences from BKPyV wild-type (WT), plasma, urine, and CSF-derived isolates. Nucleotide position 1 corresponds to the first base of the NCCR upstream of the early gene region. Asterisks indicate mismatches relative to the WT sequence, while dashes represent deletions or gaps introduced for alignment.

**Figure 3:**
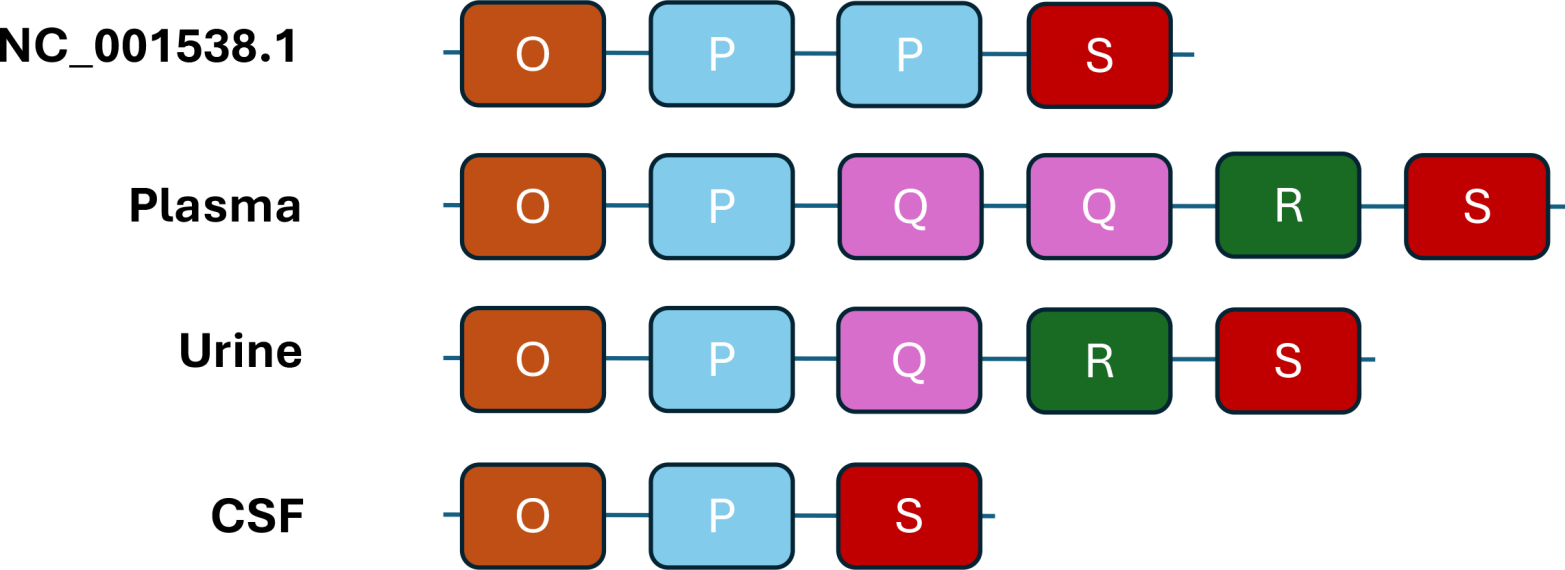
Schematic representation of the BK polyomavirus (BKPyV) NCCR structures. The Dunlop reference strain (NC_001538.1) analyzed in this study shows an O–P–P–S configuration. In the patient, the plasma-derived isolate exhibited an O–P–Q–Q–R–S arrangement with duplication of the Q block, where the first Q block was partially conserved (only the first 19 nucleotides) while the second Q block was complete, and all the remaining blocks were preserved. The urine-derived isolate displayed a classical O–P–Q–R–S structure with all blocks fully conserved. The CSF-derived isolate showed a rearranged NCCR lacking both the Q and R blocks (O–P–S, ΔQ–R variant), with the S block showing a 5′ terminal truncation of four nucleotides, whereas the O and P blocks were fully preserved. Point mutations identified by sequence alignment are reported in **Figure 2** and are not individually depicted in this schematic representation.

When comparing the non-coding control region (NCCR) sequences from plasma, urine, and CSF to the reference BKPyV Dunlop strain (NC_001538.1), we observed clear compartment-specific rearrangements involving the central enhancer region (**Figures 2**, **3**) (Martí-Carreras et al., 2020).

The plasma-derived NCCR showed a duplication of the Q block (OPQQRS), consistent with an expanded enhancer. The urine-derived sequence retained a single Q block (OPQRS) without further structural alterations. In contrast, the CSF-derived NCCR was markedly shortened, lacking both Q and R blocks (OPS). The distal T-rich/ORI and 3′ segment were conserved across all compartments.

These data indicate compartment-specific NCCR remodeling driven by enhancer block copy-number variation, most pronounced in the CSF, which may modulate viral transcriptional activity and contribute to tissue-specific adaptation.

## 4 Discussion

Few cases of BKPyV encephalitis have been reported in HSCT patients and in other immunodeficient conditions (Jun et al., 2016; Yamaguchi et al., 2024). In BKPyV encephalitis a variety of symptoms have been described including weakness, vision problems, speech difficulties, and cognitive changes, in our case, progressive cognitive and motor deficits were observed. Usually, computed tomography (CT) and MRI are nonspecific, as in our case.

In a study the diagnosis of BKPyV encephalitis was based on the detection of BKPyV DNA PCR in CSF, complemented by BKPyV PCR biopsy specimen (Stoner et al., 2002). In our case a brain biopsy was not performed due to the clinical conditions of the patient.

Based on the clinical picture, of the positive result for BKPyV PCR in CSF, and MRI suggesting primarily an inflammatory pathology it is reasonable to consider this case as a BKPyV-associated PML.

It is important to note that BKPyV DNA was detected in three different CSF samples obtained from lumbar puncture and in plasma lower value of BKPyV DNA were observed, allowing exclusion of blood contamination of the CSF. In our case BKPyV DNA was detected at levels similar to those observed in previously reported cases (10*^2–^*10{sp}3{it} {/sp}{/it}copies/mL), although the specificity and optimal cut-off values of BKPyV DNA in the CSF are unknown and clear diagnostic criteria for BKPyV encephalitis need to be established (Stoner et al., 2002; Chittick et al., 2013). Serological testing showed high anti-BKPyV and anti-JCPyV IgG levels in plasma, indicating prior exposure to both viruses, while antibodies were undetectable in CSF, consistent with lack of intrathecal synthesis. This compartmental dissociation in antibody distribution between plasma and CSF, along with the detection of BKPyV DNA and absence of JCPyV DNA in CSF, supports the hypothesis of BKPyV neurotropism. The lack of antibodies in CSF, combined with the detection of BKPyV DNA and the absence of JCPyV DNA, suggests that BKPyV was the causative agent of the PML. Although these findings support the interpretation of viral reactivation, definitive proof would require documentation of BKPyV infection prior to disease onset; therefore, primary infection occurring during profound immunosuppression cannot be completely excluded. In addition, because neither brain biopsy nor post-mortem examination were available, direct histopathological confirmation and viral detection within CNS lesions could not be performed, precluding definitive etiological attribution at the tissue level.

Our sequencing results highlight a clear intra-host variability of BKPyV across compartments, involving both VP1 (Helle et al., 2017) and the NCCR (Walder et al., 2025). At the VP1 level, we observed conserved genotype II assignment, in line with previous reports showing genotyping stability despite minor sequence heterogeneity (McIlroy et al., 2019; Morel et al., 2017). Four major genotypes of BKPyV are known: I, II, III and IV. Genotype I is the most prevalent and distributed worldwide, genotype IV is present in East Asia and Europe, whereas genotypes II and III are rarely observed; notably, genotype II has also been reported in Europe at a low frequency (Blackard et al., 2020; Jin et al., 1993; Yogo et al., 2009). In this context, the patient was born and raised in Italy, with no known ethnic or geographic background differing from the general Italian population. To date, no clear correlations between BKPyV genotype and pathology have been established and further studies are required to assess a neurotropic role for genotype II and in PML development.

The major capsid protein of BKPyV, VP1, is involved in the interaction with host cellular receptors but whether specific VP1 polymorphisms are linked to neurological sequelae of BKPyV is not clear (Neu et al., 2009; Saribas et al., 2019). The detection of compartment-specific changes in the CSF, such as the G57 insertion and N62K, is consistent with intra-patient VP1 evolution and may represent early signatures of selective pressure (McIlroy et al., 2019). This observation aligns with findings by Darbinyan et al., 2016, who reported genetic divergence of BKPyV in brain tissue from immunocompromised individuals, highlighting the potential for neurotropic variants to emerge *in-vivo*.

For the NCCR, we identified marked compartment-specific differences. NCCR rearrangements have been linked to altered transcriptional activity *in vitro* (Bárcena-Panero et al., 2012), although archetypal NCCRs have also been found in CSF isolates, indicating they are not universal markers of neurotropism. In kidney transplant recipients, rearrangements involving the Q and R blocks have been associated with enhanced viral replication and, in some cases, with more severe clinical manifestations (Virtanen et al., 2018; Liimatainen et al., 2020). In our patient, the CSF-derived isolate displayed a ΔQ–R NCCR variant, raising the possibility that similar structural alterations may facilitate viral adaptation or replication within the CNS. However, direct evidence supporting this mechanism in brain infection is currently lacking, and the functional relevance of these rearrangements in neurotropism remains to be determined. These findings suggest that BKPyV undergoes compartment-specific evolution, with VP1 mutations and NCCR rearrangements acting in parallel. This intra-host heterogeneity likely reflects the interplay of host immune pressure, replication dynamics, and tissue-specific selective forces. Further studies are needed to elucidate whether such compartmentalization influences viral pathogenicity, immune evasion, or response to therapy.

A genetic predisposition to JCPyV-induced PML is supported by the molecular diagnosis of inborn errors of immunity (IEIs) in PML patients. To date, 26 genes believed to cause IEIs and PML have been identified (Eis et al., 2025). Genetic studies are required to assess whether our patient was a carrier of a known genetic IEIs or whether an alternative genetic background might be associated with the BKPyV-induced PML. Overall, the baseline immunological vulnerability of the patients, with pre-existing bronchiectasis and history of recurrent infections, support the hypothesis of IEIs.

Mogamulizumab is a defucosylated humanized monoclonal antibody targeting the CC chemokine receptor 4 (CCR4), highly expressed on malignant T cells in cutaneous T-cell lymphoma and adult T-cell leukemia/lymphoma (Whittaker et al., 2016; Zengarini et al., 2024). Its antitumor activity is mediated primarily through enhanced antibody-dependent cellular cytotoxicity via FcγRIIIa engagement on natural killer cells, leading to efficient elimination of CCR4-positive malignant cells. In parallel, Mogamulizumab depletes CCR4-expressing regulatory T cells (Tregs), thereby modulating the host immune environment. In addition, blockade of the CCR4–CCL17/CCL22 signaling axis may impair lymphocyte trafficking and tissue homing. While these mechanisms support therapeutic efficacy, the profound reduction of Tregs and resulting immune imbalance may impair immune surveillance against latent viral pathogens. Accordingly, cases of viral reactivation and opportunistic infections, including hepatitis B virus reactivation and cytomegalovirus disease, have been reported during Mogamulizumab therapy (Wang et al., 2020). Our case suggests that a similar immune dysregulation may predispose to BKPyV reactivation and CNS dissemination, underscoring the importance of infectious monitoring in treated patients. In our case, for the first time in a Mogamulizumab-treated patient, JCPyV and BKPyV reactivations were observed, and BKPyV-associated PML was diagnosed, highlighting the increased risk of opportunistic infections. The profound lymphopenia induced by Mogamulizumab, together with Treg depletion, might predispose patients to unusual opportunistic infections, including neurotropic polyomaviruses.

Current Mogamulizumab protocols lack standardized guidelines for viral monitoring, however Mogamulizumab therapy carries a non-negligible risk of severe infections due to its immune-modulating mechanism. Our case showing the reactivation of CMV, JCPyV and BKPyV infections highlights the relevance of a closer surveillance for viral reactivations of CMV, JCPyV, BKPyV, and others such as EBV, particularly in high-risk patients. Moreover, our case suggests that BKPyV-associated PML may be an emerging, under-recognized complication in patients receiving potent T-cell-targeted therapies such as Mogamulizumab. The case underscores the need for heightened vigilance for atypical CNS viral infections, including BKPyV -associated PML, in patients treated with Mogamulizumab, especially in the presence of pre-existing immunodeficiency, since the immunological vulnerability of the patient may have further increased the susceptibility to a severe viral complication during therapy.

The monitoring could be helpful to understand whether Mogamulizumab plays a role in the development of neurotropic variants of BKPyV.

Further studies are warranted to better define prophylactic and monitoring strategies, including a genetic assessment for IEIs.

Finally, our case supports the importance of considering BKPyV infection in the differential diagnosis of immunosuppressed patients presenting with clinical symptoms of encephalopathy and undetectable JCPyV in the CSF.

In conclusion, BKPyV typically causes nephropathy in transplanted patients, although its neurotropic potential has been recognized in immunocompromised hosts. So far, rare cases of BKPyV-associated PML have been reported and our case is the first associated with Mogamulizumab treatment. This case highlights the potential role of BKPyV as a neurotropic pathogen in the setting of Mogamulizumab-induced immunosuppression. Although systematic BKPyV screening is not currently recommended, targeted testing should be considered in patients developing unexplained neurological deterioration under CCR4 blockade.

## Data Availability

The results supporting the findings of this study are included in the article and its **Supplementary Material**. Sequence data generated by Sanger sequencing were obtained from routine clinical diagnostic procedures in a single patient. Due to ethical and privacy considerations, these data are not publicly deposited but are available from the corresponding author upon reasonable request.
