# Supplementary material for: BK polyomavirus-associated progressive multifocal leukoencephalopathy following mogamulizumab therapy for erythrodermic mycosis fungoides
Source: Front Cell Infect Microbiol. 2026 Feb 4;16:1733473. doi: 10.3389/fcimb.2026.1733473 (PMC12913559; doi:10.3389/fcimb.2026.1733473)
Supplement: Supplementary file 1 [file DataSheet1.pdf]

Parameter	19/12/2024	13/02/2025	Unit
**WBC**	3.54	4.39	x10^3/µL
**RBC**	4.64	3.29	x10^6/µL
**Hemoglobin**	13.4	9.7	g/dL
**Hematocrit**	41.2	28.4	%
**MCV**	88.7	86.3	fL
**MCH**	28.9	29.5	pg
**MCHC**	32.9	34.2	g/dL
**RDW**	13	13	%
**Platelets**	264	254	x10^3/µL
**PCT**	0.25	0.28	%
**MPV**	9.6	11.2	fL
**PDW**	37.1	12.3	%
**Neutrophils %**	58.7	60.5	%
**Lymphocytes %**	29.8	27.3	%
**Monocytes %**	8.2	8.7	%
**Eosinophils %**	0.8	3	%
**Basophils %**	0.5	0.5	%
**Sodium**	140	130	mmol/L
**Potassium**	4.6	4.6	mmol/L
**Glucose**	116	102	mg/dL
**Creatinine**	0.55	0.45	mg/dL
**eGFR**	99	116	mL/min/1.73m^2
**Total Bilirubin**	0.57	1.55	mg/dL
**Direct Bilirubin**	0.14	1	mg/dL
**ALT**	27	44	U/L
**CK**	106	107	U/L
**CRP**	49.5	65.6	mg/L

Supplementary Table 1. Hematological and biochemical parameters at two time points. Complete blood count and serum biochemistry values measured on December 19, 2024, and February 13, 2025. Units are indicated in the rightmost column.

```
VP1 DNA sequence

       [1..100]
WT   : ATGGCCCCAACCAAAAGAAAAGGAGAGTGTCCAGGGGCAGCTCCCAAAAAGCCAAAGGAACCCGTGCAAGTGCCAAAACTACTAATAAAAGGAGGAGTAG
Plas : ATGGCCCCAACCAAAAGAAAAGGAGAGTGTCCAGGGGCAGCTCCCAAAAAGCCAAAGGAACCCGTGCAAGTGCCAAAACTACTAATAAAAGGAGGAGTAG
Urin : ATGGCCCCAACCAAAAGAAAAGGAGAGTGTCCAGGGGCAGCTCCCAAAAAGCCAAAGGAACCCGTGCAAGTGCCAAAACTACTAATAAAAGGAGGAGTAG
CSF  : ATGGCCCCAACCAAAAGAAAAGGAGAGTGTCCAGGGGCAGCTCCCAAAAAGCCAAAGGAACCCGTGCAAGTGCCAAAACTACTAATAAAAGGAGGAGTAG


       [101..200]
WT   : AAGTTCTAGAAGTTAAAACTGGGGTAGATGCTATTACAGAGGTAGAATGCTTCCTAAACCCAGAAATGGGGG---ATCCAGATGAAAACCTTAGGGGCTT
Plas : AAGTTCTAGAAGTTAAAACTGGGGTAGATGCTATAACAGAGGTAGAATGCTTTCTAAACCCAGAAATGGGGG---ATCCAGATGATAACCTTAGGGGCTA
Urin : AAGTTCTAGAAGTTAAAACTGGGGTAGATGCTATAACAGAGGTAGAATGCTTTCTAAACCCAGAAATGGGGG---ATCCAGATGATAACCTTAGGGGCTA
CSF  : AAGTTCTAGAAGTTAAAACTGGGGTAGATGCTATAACAGAGGTAGAATGCTTTCTAAACCCAGAAATGGGGGGGGATCCAGATGATAAACTTAGGGGCTA
                                         *                 *                   ***          *  *          *

       [201..300]
WT   : TAGTCTAAAGCTAAGTGCTGAAAATGACTTTAGCAGTGATAGCCCAGAGAGAAAAATGCTTCCCTGTTACAGCACAGCAAGAATTCCCCTCCCCAATTTA
Plas : TAGTCTAAAGCTAACTGCTGAAAATGCCTTTGACAGTGATAGCCCAGACAAAAAAATGCTTCCTTGTTACAGCACAGCAAGAATTCCACTGCCCAATCTA
Urin : TAGTCTAAAGCTAACTGCTGAAAATGCCTTTGACAGTGATAGCCCAGACAAAAAAATGCTTCCTTGTTACAGCACAGCAAGAATTCCACTGCCCAATCTA
CSF  : TAGTCTAAAGCTAACTGCTGAAAATGCCTTTGACAGTGATAGCCCAGACAAAAAAATGCTTCCTTGTTACAGCACAGCAAGAATTCCACTGCCCAATCTA
                     *           *    **               * *            *                       *  *      *

       [301..400]
WT   : AATGAGGACCTAACCTGTGGAAATCTACTGATGTGGGAGGCTGTAACTGTACAAACAGAGGTTATTGGAATAACTAGCATGCTTAACCTTCATGCAGGGT
Plas : AATGAGGACCTAACCTGTGGAAATCTACTAATGTGGGAGGCTGTAACTGTAAAAACAGAGGTTATTGGAATAACTAGCATGCTTAACCTTCATGCAGGGT
Urin : AATGAGGACCTAACCTGTGGAAATCTACTAATGTGGGAGGCTGTAACTGTAAAAACAGAGGTTATTGGAATAACTAGCATGCTTAACCTTCATGCAGGGT
CSF  : AATGAGGACCTAACCTGTGGAAATCTACTAATGTGGGAGGCTGTAACTGTAAAAACAGAGGTTATTGGAATAACTAGCATGCTTAACCTTCATGCAGGGT
                                    *                     *

       [401..500]
WT   : CACAAAAAGTGCATGAGCATGGTGGAGGAAAACCTATTCAAGGCAGTAATTTCCACTTCTTTGCTGTAGGTGGAGAACCCTTGGAAATGCAGGGAGTGCT
Plas : CCCAAAAAGTTCATGAGAATGGTGGAGGCAAACCTGTCCAAGGCAGTAATTTCCACTTCTTTGCTGTGGGTGGAGACCCCTTGGAAATGCAGGGAGTGCT
Urin : CCCAAAAAGTTCATCAGAATGGTGGAGGCAAACCTGTCCAAGGCAGTAATTTCCACTTCTTTGCTGTGGGTGGAGACCCCTTGGAAATGCAGGGAGTGCT
CSF  : CCCAAAAAGTTCATGAGAATGGTGGAGGCAAACCTGTCCAAGGCAGTAATTTCCACTTCTTTGCTGTGGGTGGAGACCCCTTGGAAATGCAGGGAGTGCT
        *        *   *  *          *      * *                             *        *

       [501..599]
WT   : AATGAATTACAGGTCAAAGTACCCTGATGGTACTATAACCCCTAAAAACCCAACAGCCCAGTCCCAGGTAATGAATACTGACCATAAGGCCTATTTGGA
Plas : AATGAATTACAGAACAAAGTACCCACAAGGTACTATAACCCCTAAAAACCCTACAGCTCAGTCCCAGGTAATGAATACTGACCATAAGGCCTATTTGGA
Urin : AATGAATTACAGAACAAAGTACCCACAAGGTACTATAACCCCTAAAAACCCTACAGCTCAGTCCCAGGTAATGAATACTGACCATAAGGCCTATTTGGA
CSF  : AATGAATTACAGAACAAAGTACCCACAAGGTACTATAACCCCTAAAAACCCTACAGCTCAGTCCCAGGTAATGAATACTGACCATAAGGCCTA------
                   **          ** *                       *     *                                   ******
```

Supplementary Figure 1. Multiple sequence alignment of VP1 from wild-type and compartment-derived BKV isolates. Multiple sequence alignment of the VP1 region from BKV wild-type (WT), plasma, urine, and CSF-derived isolates. Nucleotide position 1 corresponds to the first codon of the VP1 coding sequence. Asterisks indicate nucleotide differences relative to the WT sequence, while dashes represent deletions.

```
VP1 Protein sequence

       [1..100]
WT   : MAPTKRKGECPGAAPKKPKEPVQVPKLLIKGGVEVLEVKTGVDAITEVECFLNPEMG-DPDENLRGFSLKLSAENDFSSDSPERKMLPCYSTARIPLPNL
Plas : MAPTKRKGECPGAAPKKPKEPVQVPKLLIKGGVEVLEVKTGVDAITEVECFLNPEMG-DPDDNLRGYSLKLTAENAFDSDSPDKKMLPCYSTARIPLPNL
Urin : MAPTKRKGECPGAAPKKPKEPVQVPKLLIKGGVEVLEVKTGVDAITEVECFLNPEMG-DPDDNLRGYSLKLTAENAFDSDSPDKKMLPCYSTARIPLPNL
CSF  : MAPTKRKGECPGAAPKKPKEPVQVPKLLIKGGVEVLEVKTGVDAITEVECFLNPEMGGDPDDKLRGYSLKLTAENAFDSDSPDKKMLPCYSTARIPLPNL
                                                                *    **  *    *   * *    **

       [101..192]
WT   : NEDLTCGNLLMWEAVTVQTEVIGITSMLNLHAGSQKVHEHGGGKPIQGSNFHFFAVGGEPLEMQGVLMNYRSKYPDGTITPKNPTAQSQVMN
Plas : NEDLTCGNLLMWEAVTVKTEVIGITSMLNLHAGSQKVHENGGGKPVQGSNFHFFAVGGDPLEMQGVLMNYRTKYPQGTITPKNPTAQSQVMN
Urin : NEDLTCGNLLMWEAVTVKTEVIGITSMLNLHAGSQKVHQNGGGKPVQGSNFHFFAVGGDPLEMQGVLMNYRTKYPQGTITPKNPTAQSQVMN
CSF  : NEDLTCGNLLMWEAVTVKTEVIGITSMLNLHAGSQKVHENGGGKPVQGSNFHFFAVGGDPLEMQGVLMNYRTKYPQGTITPKNPTAQSQVMN
                        *                    **     *            *            *   *
```

Supplementary Figure 2. Multiple sequence alignment of VP1 amino acid sequences from wild-type and compartment-derived BKV isolates. Alignment of the predicted amino acid sequences of the VP1 protein (residues 1–192) from BKV wild-type (WT), plasma, urine, and CSF-derived isolates. Position 1 corresponds to the first methionine of the VP1 coding sequence. Asterisks indicate amino acid substitutions relative to the WT sequence, while dashes represent deletions.
